# Oral Manifestations of Suspected Eating Disorders among Women of 20-25 Years in Bangalore City, India

**Published:** 2014-03

**Authors:** Pallavi Vasantro Jugale, M. Pramila, Archana Krishna Murthy, S. Rangath

**Affiliations:** M.R. Ambedkar Dental College and Hospital, Bangalore, India

**Keywords:** Dentists, Eating disorders, Oral healthcare professional, Periomylolysis, SCOFF Questionnaire, Women, India

## Abstract

To detect the presence of eating disorders (EDs) and clinical findings in 20-25 years old women residing in professional college hostels in Bangalore city, India, a cross-sectional study was conducted. One hundred seventeen women of the 128 selected randomly participated in the study. SCOFF [Sick, Control, One-stone (14 lbs/6.5 kg), Fat, Food] Questionnaire was used for screening suspected cases of anorexia nervosa (AN) and bulimia nervosa (BN). Examination was done for systemic findings, extra-oral and intra-oral manifestations of EDs. Data obtained were analyzed using SPSS (version 13.0). The response rate was 71.3%, of which 42.7% were suspected to have EDs by SCOFF. Examination showed significantly higher prevalence of periomylolysis (p=0.004), dental caries (p=0.004), and tooth sensitivity (p=0.001) in suspected cases. The study succeeded at ‘case-finding’ of EDs with a significant prevalence of oral manifestations in suspected cases. Thus, dentists play a major role for early detection and prompt further referral of co-morbid disorders, like EDs.

## INTRODUCTION

Eating disorders (EDs) refer to a group of conditions characterized by abnormal eating habits that may involve either insufficient or excessive food intake, which proves detrimental to individual's physique (1). The exact cause for EDs is unknown. However, it is believed to be due to a combination of biological, psychological and/or environmental abnormalities. Some people are born with a predisposition to it, which can be brought to the surface, depending on environment and reactions to it.

EDs are mostly results of psychological factors (co-morbid disorders), personality traits, and environmental factors, like child maltreatment, social isolation, parental pressure, cultural and peer pressure. EDs are among the most common psychiatric problems that affect young women (2). It is 10 times more common in women (5-10 million affected in the USA) than in men (estimated 1 million) (3). The conditions caused by EDs impose a high burden of mortality and morbidity. Unfortunately, the diagnosis of eating disorders can go undetected (4). The American Psychiatric Association (APA) classifies 3 distinct ED diagnoses: anorexia nervosa (AN), bulimia nervosa (BN), and eating disorder not otherwise specified (EDNOS) (5).

Early detection and intervention of disordered eating behaviour can prevent or reduce serious conditions associated with EDs; systemic conditions, like dry scaly skin, constipation, dysphagia, malnutrition, decreased BMR, oesophagitis, including early oral manifestations, such as soft tissue trauma and angular cheilosis and irreversible damage, such as dental erosion and caries (6). Thus, ‘medical history is the most powerful tool for diagnosing ED’ (3).

Previous research (3,5,7) has noted that the Oral Healthcare Professionals (OHCPs) often are the first healthcare professionals to encounter patients with undiagnosed EDs because some of the initial signs and symptoms associated with EDs are found in or around the oral cavity (5). For prognosis of AN and BN, there seems to exist a general agreement that full recovery rates are in 50% to 85% range, with larger proportions of patients experiencing at least partial remission (8).

India is rapidly globalizing, with increasing exposure to Western media, leading to excessive importance to body image (9). Previous literature (10,11) has shown a growing trend of this condition among Indian women. Thus, a study with an objective to detect the presence of EDs and the clinical manifestations was undertaken in 20-25 years old women residing in professional college hostels in Bangalore city.

## MATERIALS AND METHODS

In this cross-sectional study, 128 women in the age-group of 20-25 years were selected as study participants, of whom 117 participated. The sample-size was calculated using the prevalence rate from previous studies (7,12,13); keeping the significance level at 5% (p=0.05) and CI at 95% confidence interval (CI). The sample frame was a list of all professional college hostels in Bangalore (14). Out of the 60 hostels enlisted, 10 hostels satisfied the inclusion criteria of being professional college hostels for females. Of these 10 female professional college hostels, 5 were selected for the present study.

A self-administered SCOFF Questionnaire (15) was used for screening the study participants for AN and BN. The questionnaire helped raise suspicion about the cases whether they were suspected for EDS, especially anorexia nervosa and bulimia nervosa. Suspected cases were those who gave positive answers for more than one question in SCOFF Questionnaire. The validation of the questionnaire was done, and the Cronbach's alpha obtained was 0.70. Third question of the original questionnaire was intended to know whether the reduction in weight was more than 2 stones (15). For the current study, 1 stone was converted to kg (1 stone=6.5 kg) (16). To confirm the suspicion, clinical examination was done for nine prominent conditions of ED, i.e. atrophic mucosa, ulcerations, periomylolysis (palatal erosion of maxillary anteriors), dental caries, tooth sensitivity, dental erosion (intra-oral findings), angular cheilitis, parotid gland enlargement (extra-oral findings), and Russell's finger (systemic finding). Extra-oral and systemic finding were assessed by palpation and inspection respectively. The intra-oral findings were assessed with the help of a mouth mirror. ‘Heat test’ was employed to test the tooth sensitivity. Dental caries was identified by applying the WHO criterion (modified 1997). Single examiner checked all the study participants for extra- and intra-oral examinations before undertaking the procedure. The intra-examiner kappa was 0.83. Data obtained were analyzed using Statistical Package for Social Sciences (version 13.0).

Permission was obtained from the Institutional Ethical Committee of M.R. Ambedkar Dental College and Hospital, Bangalore and the concerned hostel authorities for conducting the study. Consent was taken from every participant after explaining the purpose of the study. Only those who agreed to participate were included in the study.

## RESULTS

Of the total 128 women staying in the hostel, 117 women participated in the study. The response rate was 71.3%. The women in the study were 20-25 years of age, with a mean age of 21.9 years [SD=±1.70].

Fifty (42.7%) participants gave positive answers to more than one question in SCOFF Questionnaire and, thus, were under the suspicion of having EDs—AN or BN (Table 1)

**Table 1.** Response to the SCOFF Questionnaire

Criterion	n (%)
<2 positive answers (unsuspected)	67 (57.26)
>2 positive answers (suspected)	50 (42.73)

Table 2 shows the distribution of the clinical findings of AN and BN to the suspected and unsuspected groups determined by the SCOFF. Out of the nine clinical findings, three showed significant correlation with SCOFF [(periomylolysis (p=0.004), dental caries (p=0.004), and tooth sensitivity (p=0.001). Russell's finger and parotid gland enlargement were not seen in any of the participants (n=0)].

## DISCUSSION

The present study on women aged 20-25 years and residing in professional college hostels succeeded in finding suspected cases of EDs. The instruments used and the methodology applied helped us achieve our objective to detect the presence of EDs, and the clinical findings showed significance for three conditions of periomylolysis, dental caries, and tooth sensitivity, which are few of the prominent manifestations of EDs concurrent with other studies (17-20).

SCOFF proved to be a reliable instrument in differentiating the suspected and the unsuspected cases of AN and BN. Our study showed 42.7% of suspected and 57.3% of unsuspected cases by the use of SCOFF Questionnaire as in previous studies (3,21). SCOFF Questionnaire used in this study is simple, memorable, and easy to apply and has scores. Many studies have shown it to be one of the promising screening tools in primary-care settings (3,5). The five questions in SCOFF Questionnaire are appropriate to raise suspicion for eating disorders—anorexia nervosa and bulimia nervosa (15).

**Table 2.** Distribution of oral manifestations in subjects

Condition	Suspected (n=50)		Unsuspected (n=67)		p value
	Present n (%)	Absent n (%)	Present n (%)	Absent n (%)	
Atropic mucosa	9 (18)	41 (82)	8 (11.9)	59 (88.1)	0.287
Ulceration	5 (10)	45 (90)	7 (10.4)	60 (89.6)	0.971
Periomylolysis	16 (32)	34 (68)	7 (10.4)	60 (89.6)	0.004*
Russell's finger	0	50 (100)	0	67 (100)	-
Dental caries	39 (78)	11 (22)	37 (55.2)	30 (44.8)	0.004*
Angular cheilosis	1 (2)	49 (98)	2 (2.9)	65 (97.1)	0.779
Tooth sensitivity	28 (56)	22 (44)	14 (20.9)	53 (79.1)	0.001*
Cervical abrasion	4 (8)	46 (92)	2 (2.9)	65 (97.1)	0.193
Parotid gland enlargement	0	50 (100)	0	67 (100)	-

*denotes significance

EDs prominently show medical problems which have been well-described in literature for many years (3,5,22). However, the effects of EDs on oral tissues are the first signs and symptoms to be noticed. The six intra-oral findings considered in this study are the most commonly-encountered conditions comparable to previous studies (5,22). Typical pattern of erosion on palatal surface (periomylolysis) is the prominent and diagnostic finding (p=0.004) of the present study as in the studies done by Little and DeBate (22,23). Periomylolysis is generally caused by vomiting type of eating disorders. The present study does not depict the above cause for periomylolysis since none of the participants gave positive answer to the SCOFF question “Do you make yourself sick because you feel uncomfortably full?” Thus, periomylolysis can be attributed to excessive intake of acidic foods and drinks and maintaining the drink in mouth for long time (24,25).

Dental caries was also significant (p=0.004) in the present study. Other studies have also shown dental caries to be one of the significant findings for EDs (17-19) but the cause of dental caries being multifactorial, it's presence cannot be entirely attributed to EDs. Parallel factors to be considered are individual's oral hygiene, cariogenicity of the diet, malnutrition, genetic predisposition, fluoride experience during tooth development, and ingestion of certain types of medication. Similar inconsistent findings between ED and dental caries have also been found in earlier research (22). Tooth sensitivity, detected by ‘heat test’, was found significant in our present study. Tooth sensitivity can be due to periomylolysis or dental caries. The cause of tooth sensitivity cannot be implicated entirely to eating disorders but can be said to be the secondary manifestations to dental caries, cervical erosion, periomylolysis, in EDs.

Women aged 20-25 years were chosen for the study since most studies done direct us towards the similar sex predilection for EDs (3). A study done by T. Srinivasan in Chennai, India, showed 10 times more prevalence of EDs in women (10). Furthermore, EDs begin around puberty but may appear later, usually by middle age (23).

The study was conducted in professional college hostels where lifestyle of women has an impact on their perceptions of appearance, their eating habits, and schedule. In professional colleges, the stress-load being more, dysfunctional eating tendencies are at higher end compared to general population (26). Dysfunctional eating is considered an epidemic in the USA amongst adolescent and young women. Thus, this is associated with the highest rates of morbidity and mortality than any other mental health diagnosis (5). Early diagnosis and at an early age is the best and ideal method to deal with the EDs (3).

Early detection and intervention plays a key role in the recovery of eating disorders. Dental practitioners are instrumental since they are often the first health professionals to identify signs and symptoms of disordered eating (2,22). This ‘case-finding’ role has been well-established and accepted by dentistry (23).

India is a fast-developing country, with increased globalization in the last decade. India is increasingly exposed to Western media, and there has been greater emphasis on body image and eating disturbances in Indian adolescent and young women. An increasing trend of prevalence was noted from none (10) to 1.25% (11) in Indian populations. However, the trends in EDs in India do not meet full criteria of clinical features for AN and BN of established instruments as in Western countries (7). Thus, it can be concluded that the EDs in India are currently in evolution phase presenting ‘archaic’ form—infrequent, mild, and benign in nature (10).

Using the SCOFF Questionnaire to seek the suspected ED cases and determining the presence of oral manifestations, the problems associated with sample-size, more promising criterion, and tool for diagnosing the EDs remain the caveat.

### Conclusions

The present study raised suspicion about eating disorders among the participants through SCOFF Questionnaire. Additionally, positive clinical manifestations were found to be significant in the suspected group. Thus, the study directs us to the arena of case-finding of EDs, which usually goes undetected. Further theory-based research is needed to explore the precursors and parameters of eating disorders for “early detection and specific protection” by dental care providers. The identification of these parameters can facilitate the development of enhanced dental curriculum and, subsequently, primary and secondary prevention of eating disorders.

## ACKNOWLEDGEMENTS

We acknowledge the support and guidance of Dr. G.K. Umashankar (Reader), Dr. M.K. Vanishree (Senior Lecturer), and Dr. Aditi Verma (Senior Lecturer) of the Department of Public Health Dentistry, M.R. Ambedkar Dental College and Hospital, Bangalore, India.
